# Peer Review of “Perception and Impact of White Spot Lesions in Young People Undergoing Orthodontic Treatment and Their Guardians: Protocol for a Mixed Methods Study”

**DOI:** 10.2196/80140

**Published:** 2025-09-12

**Authors:** Abdolreza Jamilian

**Affiliations:** 1 The City of London Dental School, University of Greater Manchester, Bolton, United Kingdom

**Keywords:** orthodontics, white spot lesions, fixed appliances, dentistry

*This is a peer-review report for “Perception and Impact of White Spot Lesions in Young People Undergoing Orthodontic Treatment and Their Guardians: Protocol for a Mixed Methods Study.”*

## Round 1 Review

1. The abstract of this paper [1] must be revised. “Several studies explore the prevention and/or treatment of WSL” is inappropriate for the abstract.
2. The Methods section describes the mixed methods approach well, but the recruitment process could be elaborated. For example, how will convenience sampling be conducted to avoid bias? Including qualitative and quantitative data is well-justified, but there is no mention of how the two datasets will be integrated into the analysis. More details on how the qualitative data will expand upon the quantitative findings would strengthen the methodology.
3. The sample size rationale is explained well, though stating why a power analysis is unnecessary for a descriptive study could prevent confusion.
4. In the Results section, it would be useful to clarify how data from questionnaires and interviews will be compared and whether there is an expectation of divergence between parent and child responses.
5. The Limitations section acknowledges some important aspects, such as recruiting from only one hospital, but it does not address potential biases in self-reported data. There is also no mention of how the study will address participants’ potential reluctance to report negative experiences due to social desirability bias. Expanding on these limitations and how the study will mitigate them would improve transparency.
6. The potential psychological impact of white spot lesions could be expanded upon in the Discussion, especially regarding how white spot lesions may affect patient compliance and satisfaction posttreatment.
7. To expand the Discussion, the following article must be cited: Jamloo H, Majidi K, Noroozian N, et al. Effect of fluoride on preventing orthodontics treatments-induced white spot lesions: an umbrella meta-analysis. *Clin Investig Orthod*. April 19, 2024;83(2):53‐60. [doi: 10.1080/27705781.2024.2342732]
